# Dynamically Forming Electrides Based on Ionic Conductors: Physicochemical Foundations, Nanoengineering, and Prospects

**DOI:** 10.3390/ma19204349

**Published:** 2026-10-16

**Authors:** Alexandr Despotuli, Alexandra Andreeva, Anastasia Despotuli, Viacheslav Kazmiruk

**Affiliations:** Institute of Microelectronics Technology and High Purity Materials, Russian Academy of Sciences (IMT RAS), 142432 Chernogolovka, Russia; andreeva@iptm.ru (A.A.); despotuli.aa@iptm.ru (A.D.); kazmiruk@iptm.ru (V.K.)

**Keywords:** nanoionics, anionic electrons, dynamically forming electrides, solid electrolytes, superionic conductors, degenerate Fermi liquid

## Abstract

**Highlights:**

- Dynamically forming electrides under core descriptor *φ*_MH_ − *φ*_MG_ = Δ*φ*_HG_ > 0.
- Dynamically forming electrides: from *F*-centers to degenerate Fermi liquids.
- New classes of non-stoichiometric electrides based on ionic conductors.
- Expanding the chemical electride space to a chemical–technological domain driven by interfacial spontaneity (Δ*G* < 0).
- Transition to dynamic non-equilibrium processes at nanoscale.
- Great opportunities for application of non-stoichiometric electrides.
- Synergy of electrides–berthollides with solid-state ionics and nanoionics.

**Abstract:**

In modern science, the field of electrides has emerged as a highly active research frontier. An intense surge of interest in these materials—phases with high concentrations of anionic electrons—is driven by their promise for vast applications and unique properties. Non-stoichiometric electrides—materials hosting interstitial electron densities exceeding those in *F*-centers—remain scarce. Traditional computational screening relies on idealized static structures at 0 K, overlooking such materials. Here, we overcome this limitation by introducing a nanoionics paradigm in the field. Dynamically forming non-stoichiometric electrides arise under thermodynamically favorable conditions (Δ*G* < 0) in non-equilibrium nanosystems based on silver/copper halide solid-state ionic conductors. We demonstrate that stable electride phases form when the ionization potential of mobile host cations exceeds that of incident guest metal atoms; the core descriptor of these processes is *φ*_MH_ − *φ*_MG_ = Δ*φ*_HG_ > 0. The resulting materials exhibit carrier densities exceeding 10^21^ cm^−3^—well above the Mott degeneracy criterion—consistent with degenerate Fermi liquid behavior, pending direct transport confirmation. These materials remain stable in vacuum or inert gas at ambient pressure. This work establishes a generalizable framework for expanding the chemical space of electrides beyond the traditional field and creates synergy between the domain of electrides–berthollides and solid-state ionics/nanoionics/spintronics.

## 1. Introduction: Physics of the Electride State, History of Development, and Search Routes for Electride Phases

In modern condensed matter physics and chemistry, the search, design, and controlled synthesis of inorganic and organic electrides are experiencing an intensive surge [1,2,3]. Intensive research activity in this field is driven by the unique electronic structure of electride phases, low electron work function values, and prospects for practical application in heterogeneous catalysis, thermal field emission, and advanced spintronics [1,2,3].

By definition, inorganic electrides are a class of crystalline compounds. In these materials, the interstitial voids are populated by localized interstitial electrons serving as anions, evidenced by distinct electron density maxima within the lattice [1,2,3]. The fundamental physical archetype of the electride state is rooted in the classic color center (*F*-center) of Robert Pohl’s Göttingen school [4], a historical model, in which an electron resides within a vacancy of the crystal’s anionic sublattice.

In electrides, the concentration, spatial localization, and dynamics of electrons can vary significantly, ranging from dilute, practically non-interacting *F*-centers to a strongly correlated, high-density degenerate electron liquid [5]. The shift in terminology from *F*-centers to the electride concept is driven by a qualitative change in the role of localized electrons as their concentration increases. At low concentrations, isolated *F*-centers behave as conventional point defects that do not perturb the host (*H*) lattice’s macroscopic symmetry, interatomic distances, or the thermodynamic state of the crystal. In contrast, within the electride paradigm, electrons localized inside of the voids of the *H*-lattice constitute a fundamental structural component that contributes to the thermodynamic phase stability and determines the macroscopic properties of the material.

**Figure 1:**
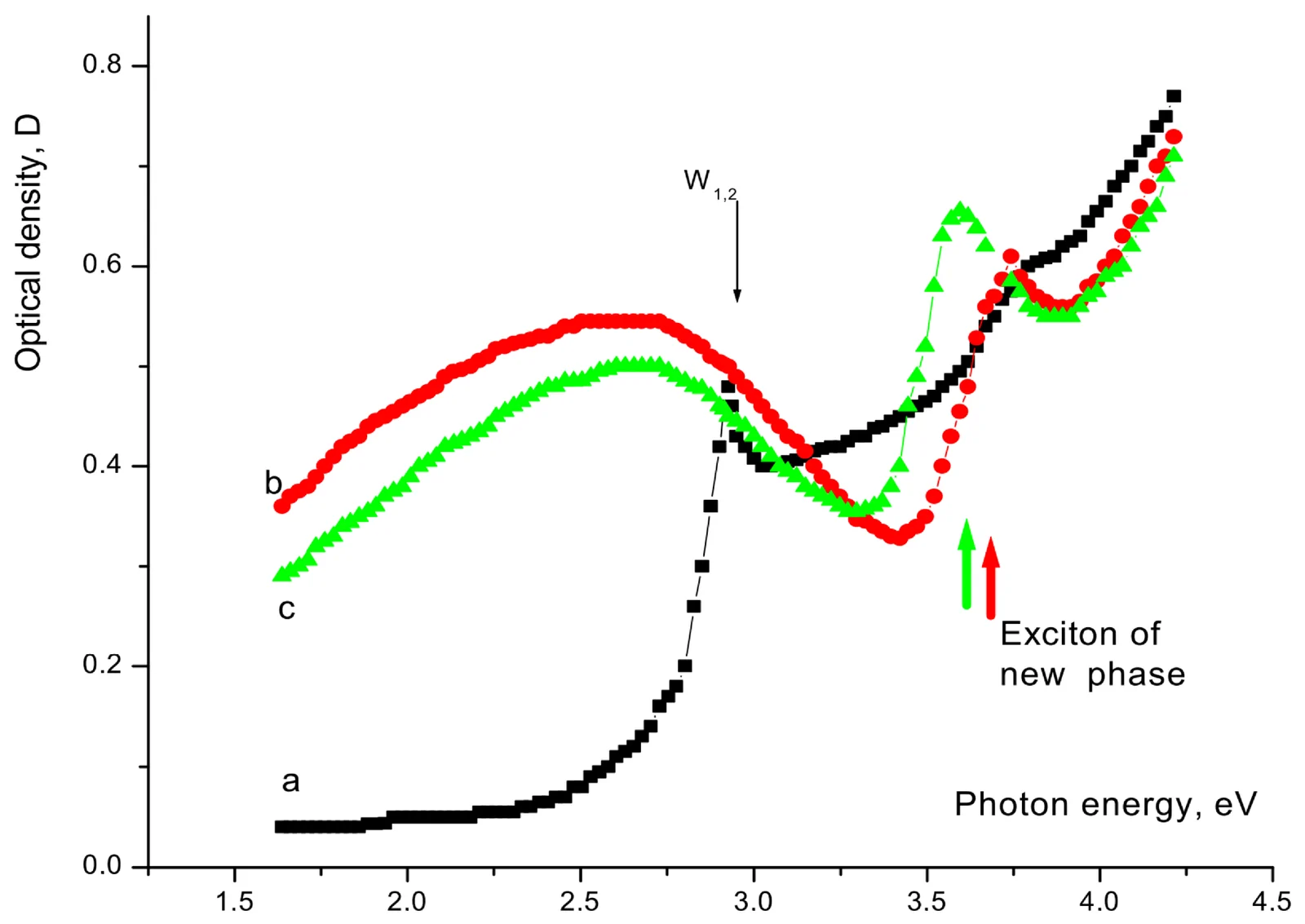
Optical density spectra *D*(hν) in the AgI/Nd thin-film nano-physicochemical system (as defined above) at 300 K: (**a**) initial film of β-AgI with a thickness of 0.05 μm; (**b**) sample after vacuum deposition of a 0.007 μm thick Nd film at 300 K onto β-AgI-film (**a**); (**c**) electride spectrum after 5 h of exposure to ambient air.

According to our data, the regime of independent *F*-centers is realized at densities *N_F_* up to ∼10^19^ cm^−3^. This conclusion is drawn from the optical absorption behavior shown in Figure 1, where a weak dependence of the *F*-center absorption band maximum on *N_F_* is observed in the advanced superionic conductor α-RbAg_4_I_5_. Because the *F*-center physics in this halide is analogous to that in the alkali halides studied by R. Pohl’s school, our data extend the upper limit of independent *F*-center behavior from ∼10^17^–10^18^ cm^−3^ (typical of rigid alkali-halide lattices) to ∼10^19^ cm^−3^. Physically, this extension is consistent with the nature of the superionic matrix: the high concentration of mobile Ag^+^ ions (∼10^22^ cm^−3^) provides strong screening of the Coulomb interaction between *F*-centers, thereby preserving their independence up to densities far exceeding those typical of rigid lattices. The transition to a degenerate state begins at densities somewhat below 10^20^ cm^−3^ and is governed by the Mott criterion *a_B_*^∗^·*n_c_*^1/3^ ≈ 0.25, where *a_B_*^∗^ = *a_B_*·*ε*/(*m*^∗^/*m_e_*) is the effective Bohr radius. For moderate effective masses (m^∗^ ∼ m_e_) and dielectric constants typical of the host matrices (*ε* ∼ 5–10), the critical density falls in the range of 10^20^–10^21^ cm^−3^, and the measured interstitial electron densities exceeding 3 × 10^21^ cm^−3^ satisfy this criterion.

It should be emphasized that the concentration estimate obtained via Smakula’s formula for *F*-centers is expected to underestimate the true density at such high concentrations, as the formula assumes non-interacting centers and does not account for the overlap of electronic wave functions that occurs as the Mott density is approached. In the heavy fermion regime (*m*^∗^ ≫ *me*), the Mott threshold shifts to higher densities—but a large effective mass itself arises from hybridization and delocalization of interstitial electrons, presupposing that the system is already on the degenerate side of the transition. Thus, the measured densities are consistent with the degenerate regime regardless of the effective mass regime.

Historical approaches to the controlled synthesis of electride phases evolved through several key milestones. In 1983, J. Dye pioneered the synthesis of cryogenic, thermally unstable organic electrides based on crown ethers [6]. A major breakthrough occurred in 2003 with the discovery of the first room temperature kinetically stable (although Δ*G* > 0 at formation) inorganic oxide electride based on a framework calcium aluminate mineral, mayenite (12CaO·7Al_2_O_3_ [7]). Structurally, the extra framework oxygen ions confined within sub-nanometer voids are partially or completely replaced by electrons. This yields a zero-dimensional (0D) electride exhibiting stability against ambient oxidation, the first demonstration that localized electrons can remain stable at room temperature.

The next conceptual stage of development is associated with discoveries in high-pressure physics. These studies experimentally demonstrated that elemental metallic sodium, under heavy mechanical compression at pressures exceeding 100–200 GPa, can transition into an optically transparent insulating electride phase. Experimentally, this approach relies on the diamond anvil cell technique [8].

To date, three main directions for electride screening can be identified, two of which are developing intensively, while the third remains virtually unknown. The first route involves ultrahigh pressures; this route relies on a semi-empirical rule: the ability of a chemical element to expel valence electrons into lattice interstices under pressure is directly related to its tendency to lose electrons, meaning elements with lower first ionization potentials more readily undergo this transition. This rule serves as a baseline guide determining the propensity of a substance to form an electride state under conditions of severe spatial confinement of electron orbitals. Such calculations require resource-intensive supercomputer-based crystal structure prediction methodologies [9,10].

The second route is represented by the concept of static screening, which involves the search for structurally porous crystalline phases [11,12]. The physical foundation of this approach relies on the geometric void engineering of the free space within the host *H*-lattice [11,12]. Specialized topological algorithms perform automated screening of crystallographic databases to identify stable frameworks with empty sub-nanometer voids or one-dimensional conduction channels (as observed in the 1D electride Y_5_Si_3_). The baseline criterion for such selection is the strict requirement that the calculated structure lies on the minimum energy surface—the static convex hull at a temperature of 0 K, which completely precludes the consideration of dynamic thermal fluctuations and framework lability [11,12,13].

The current frontier in electride discovery is represented by a screening algorithm [14] that utilizes neural network models to generate crystal structures, followed by convex hull verification and Electron Localization Function (ELF) analysis. The quantitative metrics of this computational approach underscore its high resource requirement. Starting with the generation of a vast structural ensemble, ~10^5^ hypothetical crystal structures, the computational algorithm progressively narrowed down the candidates through a multi-stage selection process, with the terminal DFT-based relaxation phases demanding substantial expenditure of high-performance computing (HPC) core hours. At the output of this computational funnel, the number of absolutely stable inorganic electrides lying strictly on the thermodynamic minimum line at 1 atm pressure was reduced to merely 13 compounds across the entire global database [14]. Of these, actual experimental synthesis has been achieved for only a few isolated objects. Such an outcome stems directly from the conceptual incompleteness of the paradigm of static geometric screening of fixed unit cells.

Statistical analysis of the results from high-throughput quantum–chemical screening of Inorganic Crystal Structure Databases (ICSD) allows for evaluating the true efficiency and the actual yield of physically stable electride phases obtained via automated discovery techniques. While the total array of crystal structures subjected to supercomputer analysis ranges between 10^5^ and 10^6^ compounds, the number of real, experimentally synthesized inorganic compounds lying on the static convex hull and exhibiting macroscopic stability in the electride state at 1 atm and 300 K is well below 10^2^ [15,16,17]. Consequently, the final yield of physically realizable stable structures proves to be critically low.

A conceptual breakthrough in the systematization of electrides was achieved in [1], which proposed a continuous spectrum of electride systems categorized into three major classes. The first class comprises electron-rich electrides, in which the cationic framework carries an excess formal charge compensated by electrons within the structural voids. This class also encompasses non-stoichiometric, anion-deficient oxide electrides of variable composition (berthollides), where the density of the interstitial electrons is continuously tuned by the degree of extra-framework oxygen extraction during reduction annealing. Studies on defective oxides [18] established that the enthalpy of this process is remarkably high, with Δ*H* = 360 kJ/mol. Such a large energy barrier limits the generation of high electron gas concentrations.

The second class consists of electron-neutral (neutral) electrides, where the metallization of interstitial voids occurs due to strong orbital hybridization with the free space of the unit cell. The third class includes electron-deficient electrides, in which the Coulomb wells of the interstitial channels expel electrons from the atomic orbitals of metalloids into the interstitial voids (as exemplified by Y_2_LiSi_2_).

The dynamically forming non-stoichiometric electrides of variable composition, e.g., ([RbM_x_Ag_4−3x_I_5−3x_]^3x+^·(e^−^)_3x_) type, described in this paper should occupy a special place in the classification [1]. This third route, representing a fundamental alternative to the first two approaches (which rely either on extreme mechanical compression or static framework analysis at 0 K), unfolds upon transitioning to the nanoscale design of latent multicomponent systems based on solid-state ionic conductors [19,20,21,22,23,24]. The primary conceptual difference and advantage of the third route lies in the ability to “bypass” the enthalpy barrier [18] at 300 K. In these processes at ambient pressures of 1 atm, the density of interstitial anionic electrons (denucleated electrons) is governed by phenomena occurring at atomically clean heterointerfaces. Then, all reactions proceed under the control of the difference in ionization potentials between the metal atoms of the host *H*-lattice and the guest *G*-metal, which acts as the donor of anionic electrons:

*φ*_MH_ − *φ*_MG_ ≡ Δ*φ*_HG_ > 0 (1)

where *φ*_MH_ is the first ionization potential of the metal M atoms residing as ions within the *H*-crystal and *φ*_MG_ is the first ionization potential of the *G*-metal atoms whose ions enter the *H*-crystal. In studies [19,20,21,22,23,24], Ag^+^ and Cu^+^ act as the *H*-ions. Under condition (1), the incoming flux of the donor metal generates high-density electrons localized within the halide vacancies, whereas the structural relaxation of the crystal framework is driven by a positive Cauchy lability factor.

In the series of experimental studies on the synthesis and properties of new nano-physicochemical systems with a high density of denucleated electrons summarized in this work, the physical feasibility of a wide series of ionic–electronic condensed electride media stable at 300 K was demonstrated [19,20,21,22,23,24]. These dynamically forming electrides are based on silver and copper halides acting as *H*-matrices. Within this approach, more than 10 different *G*-metals were introduced into the chemical space of the reactions. These included rare earth elements (Sm, La, Ce, Nd, Tb, Tm, Yb, Lu, Sc, Y), aluminum (Al), paramagnetic manganese (Mn), as well as Fe and Ni, which are inactive or poorly active with respect to the utilized halides.

The threshold-free (barrierless) character of the interfacial charge transfer requires clarification, as it constitutes a defining feature of the dynamically forming electride mechanism, which distinguishes it fundamentally from conventional solid-state reactions.

*H*-matrices—superionic conductors—differ fundamentally from rigid classical crystals. In the solid superionic conductors studied here, the cationic sublattice is structurally disordered and exists in a liquid-like, continuously fluctuating state. The initial boundary layer of an ionic conductor *H*-matrix is structurally disordered due to space charge effects (a concept established in the foundational work of Kurt Lehovec and now recognized as a basic fact of nanoionics) already prior to *G*-metal deposition. Critically, at the very first moment of deposition, as the incident flux of *G*-metal atoms encounters this inherently disrupted surface, the *G*-atoms transfer their valence electrons into pre-existing surface halogen vacancies (created by the Frenkel mechanism). This generates a highly nonequilibrium interfacial electron–ion condensate consisting of halide ions, newly formed cations, and solvated electrons within which the static Madelung electrostatic invariants of the bulk crystal are completely destroyed from the very onset of the process.

Under these strongly nonequilibrium conditions of the near-surface plasma, the gas-phase atomic descriptor Δφ_HG_ > 0 functions as an operationally sufficient energy selector for the initial charge transfer act. As the reaction propagates into the bulk, these fluctuating surface states evolve: native anions are progressively displaced and systematically replaced by interstitial coreless electrons, forming a true bulk electride phase.

The discriminating power of the descriptor Δ*φ*_HG_ > 0 is confirmed by an extensive empirical base comprising approximately 100 independent experiments performed with a broad set of G-metals. In every case, without exception, interfacial mass and charge transfer either proceeded efficiently or was entirely absent in predictable correspondence with the selector. *G*-metals with Δ*φ*_HG_ < 0—including not only Fe and Ni but also Au—remained completely inactive at the nano-interface, whereas elements satisfying Δ*φ*_HG_ > 0 (Mn, Nd, Sm, Yb, and others) systematically generated stable phases with high concentrations of *F*-centers.

The condition Δ*φ*_HG_ > 0 determines the possibility of the *G*-metal penetrating into the *H*-matrix and acts as a clear descriptor for the formation of electride phases. Studies [19,20,21,22,23,24,25] have demonstrated the feasibility of generating a virtually continuous spectrum of electride states in nano-physicochemical systems. The utility of the synthesis approach under consideration lies in the vast combinatorial capacity of its chemical space. On the other hand, this approach reveals vast classes of condensed media that are fundamentally resilient to standard high-throughput geometric AI-driven screening.

Scientific novelty: The present work introduces a new class of dynamically forming electrides, whose formation proceeds at 300 K as a non-equilibrium self-organized process driven by an interface spontaneity drop in Gibbs free energy (Δ*G* < 0) within open nanosystems based on solid-state ionic conductors. The primary contribution of this work is the introduction of the nanoionics–electride paradigm achieved through the systematic reinterpretation of the authors’ historical experimental dataset (1997–2003). Within this framework, it is demonstrated how the magnitude of a fundamental control parameter—defined as the difference between the first ionization potentials of the host and guest metals (Δ*φ*_HG_)—dictates the non-equilibrium evolution pathway and multi-stable trajectories of dynamically forming electrides. By conceptualizing these historical figures under a unified framework, this work provides a breakthrough paradigmatic contribution that bridges fast ion kinetics and interstitial electron localization. This conceptual connection is inherently absent from the previous literature and remains completely inaccessible to conventional high-throughput static database screening at 0 K.

New criteria for targeted screening are proposed, and the range of matrix hosts suitable for the synthesis of dynamically forming electrides is expanded. The universal possibilities for the formation of electrides (stable at normal pressures of 1 atm) within inorganic and organic matrices of ionic conductors have been demonstrated. Accounting for the novel descriptors has the potential to advance existing high-throughput computational screening methods for electride materials.

The research aims to achieve a paradigm shift: it redirects the focus away from both exotic electrides stabilized under gigantic pressures and the screening for electrides within pre-defined, rigid crystal structures to identifying matrices for dynamically forming electrides.

## 2. Materials, Methods, and Optical Properties of Modified Ionic Conductors: The Discovery of Latent Electride Phases

Optical absorption and X-ray diffraction data and results [22] provide experimental validation for the descriptor of Δ*φ*_HG_ > 0, defined as the difference between the first ionization potentials of the *H*-matrix metal and the incorporated *G*-donor metal, such as trivalent M_G_^3+^:3e^−^, during the search, design, and synthesis of electrides. The reported data originate from in situ experiments performed at 300 K under high-vacuum conditions. We investigated the interface formation between vapor-deposited *G*-metal layers (M_G_ = Sm, La, Ce, Nd, Al, etc.) and *H*-polycrystalline silver halide films (α-RbAg_4_I_5_, *β*/*γ*-AgI, CuI, and α-CsAg_4_Br_3−x_I_2+x_).

Here, we introduce the term “nano-physicochemical system” to designate a specific class of nano-heterostructures formed by vacuum deposition of metallic vapor onto a thin film of an ionic conductor (e.g., AgI). In such systems, spontaneous low-temperature interfacial reactions and cation exchange occur, leading to the dynamic formation of electride phases.

The advanced superionic conductor α-RbAg_4_I_5_ is a classic object in solid-state physics and chemistry characterized by a three-dimensional network of fast ionic transport channels at the unit cell level. This architecture provides exceptional ionic conductivity and unique functional properties. In our experiments, the thickness ratio of the metal (*L*_M_) to the halide layer (*L*_Hal_) ranged from approximately 1:100 to 1:10. Atomic concentration analysis based on the *L*_M_/*L*_Hal_ ratios indicates that the modified matrix contains up to 25 rare earth (RE) metal ions per 100 silver ions (at the maximum). Given the trivalent state of the RE elements (+3), this corresponds to approximately 75 anionic electrons per 100 initial iodine ions in the host lattice.

Solid electrolyte films were obtained through thermal vacuum evaporation of halide mixtures. Two stages of vacuum equipment were employed. In the first stage (until 1999), a VUP-4 system with a liquid nitrogen trap was used, providing a base pressure of no higher than 2 × 10^−6^ Torr. In the second stage (until 2003), a USU-4 universal ultra-high vacuum system, also equipped with a liquid nitrogen trap in the vacuum chamber and a magnetron discharge pump, was employed, achieving a base pressure of ~10^−7^ Torr. Notably, the highest concentrations of *F*-centers were obtained on the USU-4 system, underscoring the critical role of ultimate vacuum quality in the formation of the electride phase. Wurtzite *β*-AgI modification samples were prepared on quartz and sapphire substrates at room temperature; zinc-blende *γ*-AgI samples were prepared at ~80 °C. The deposition of 3d and 4f rare earth (RE) metals onto AgI films was performed through thermal evaporation and laser ablation methods. Optical absorption spectra of samples synthesized through different methods were identical, confirming the method independence of the observed spectral features.

It was found that a clean, oxide-free interface between the silver halide film and the RE metal layer is the critical factor for achieving high RE concentrations in the samples. At a preliminary calibration stage, the thickness of both AgI and RE films was determined using a combination of a piezoelectric quartz crystal monitor and a mechanical profilometer to a precision of ~3 nm. After deposition, samples were kept in the vacuum chamber for approximately 20 h, whereupon samples were stored in air. Films were solid and transparent (RbAg_4_I_5_, AgI) and had weak coloring (RbAg_4_I_5_-RE, AgI-RE). Optical absorption spectra of the films were recorded on Specord M-20 and M-40 spectrophotometers. Time distance between samples moving away from the vacuum chamber and recording optical spectra varied from some hours to days and even weeks (AgI-Tb system [23]). For long-term storage, exiccator with silica gel was used. No visual destruction of the samples based on unaided eye visualization, such as opacification or moisture, was discovered for AgI-lanthanide and AgI-Al films, in contrast to AgI-Mn deposition, which demonstrated phase layering after some weeks in air.

XRD analysis was performed using the Debye–Scherrer method with a Co anode (Kα) on samples of ~0.2 μm thickness [20,21,22,23].

**Figure 2:**
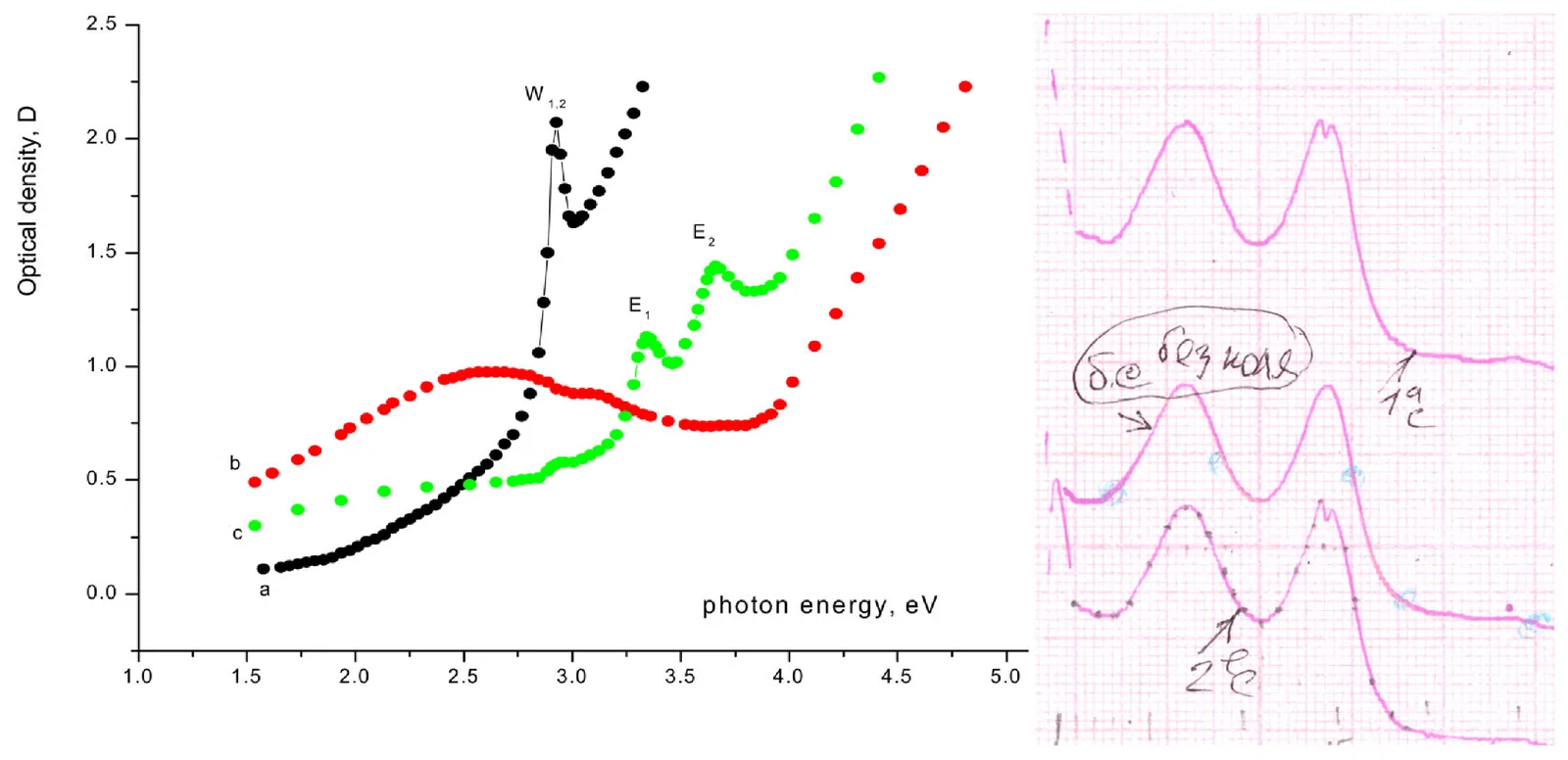
Optical density spectra *D*(*hν*) of the AgI/Mn nano-physicochemical system at 300 K, featuring an initial 0.19 μm *β* -AgI film (**a**), the sample after 0.019 μm Mn deposition (**b**), and the spectrum after 14 days of atmospheric exposure (**c**) [21]. Right panel (inset): previously unpublished archival data obtained in 2003 showing the giant Zeeman splitting of the E_1_ exciton in an Ag_2_ MnI_4_ film (composition confirmed by X-ray fluorescence analysis in an electron microscope) under a magnetic field *B* = 0.85 T at 293 K, corresponding to an effective *g*-factor of approximately 890.

Figure 1 and Figure 2, together with the accompanying excerpts from the main text, were originally reported in Ref. [21]. This preliminary communication, titled “INSERTION OF TRANSITION, RARE EARTH AND ACTINOID ELEMENTS INTO AgI AND CuI,” was deposited on the ChemWeb preprint server in 2002 (http://preprint.chemweb.com/inorgchem/0208001). The 2002 paper investigated the evolution of optical spectra, reporting significant spectral transformations as the *F*-center concentration varied. While isolated *F*-centers themselves do not constitute a true electride phase, the transition from traditional defect terminology to the electride concept reflects a qualitative change in the role of localized electrons with increasing density.

In the analyzed optical spectra, the edge exciton peak serves as an indicator of the state of the electride phase state. The quenching of this excitonic feature reflects the physical process wherein ionic bonds are superseded by interstitial electrons, marking the onset of the formation of an interacting anionic electron state. Consequently, the evolution of optical spectra enables direct monitoring of the formation and evolution of electride phases.

The applicability and feasibility of specific diagnostic methods for inorganic electrides based on silver and copper halides require consideration of a combination of physicochemical properties, processing conditions, and geometric parameters of the thin-film objects under study. In our case, the matrices are sensitive to electron and UV irradiation, and the electride films produced discolor in ambient atmosphere due to oxide formation. The films have a typical thickness on the order of 0.1–0.2 μm (∼2 · 10^−5^ cm) and a characteristic substrate area of ∼1 cm^2^. Under these specific laboratory conditions, obtaining reliable signals from electron paramagnetic resonance (EPR), X-ray photoelectron spectroscopy (XPS), or direct electrical transport measurements—which entail even brief exposure of the films to ambient atmosphere—encounters physical and instrumental limitations that render surface-sensitive and macroscopic measurements practically unfeasible.

The findings of the archived paper [21] are highly relevant to the current era of electride research. However, the preprint server hosting this work is no longer operational. Following established scientific practice for data preservation, we reproduce herein these pioneering results regarding the discovery, synthesis, and properties of dynamically forming electrides.

Figure 1 illustrates the evolution of optical absorption spectra in the *β*-AgI/Nd nano-physicochemical system at a film thickness ratio of *L*_M_/*L*_Hal_ > 0.1. As the electride phase forms in the AgI/RE system (at 300 K), the native edge exciton peak (in the 3 eV region) is completely quenched and a new resonance emerges, blue-shifted by 0.6–0.8 eV. Concurrently, a new broad absorption band appears with a maximum at approximately 2.6–2.7 eV. The observed short-wavelength (“blue”) shift of both absorption bands relative to the bands of *β*-AgI/Nd (at interstitial electron concentrations of 3·10^20^ cm^−3^) corresponds to the stable existence of a qualitatively new electride phase that remains stable at 1 atm. In an open atmosphere, this electride phase gradually loses its anionic electrons due to a chemical reaction of the metal guest with oxygen. The behavior of the optical density *D*, resembling that shown in Figure 1, indicates that new phases with a high concentration (~10^21^ cm^−3^) of Nd^3+^ or Sm^3+^ ions and corresponding high concentrations of *F*-centers are formed. 

The relationship between the exciton band and the broad *F*-center band is qualitatively straightforward, as both features depend on physical processes associated with particles residing at iodine positions. If the iodine concentration at these positions decreases (increases), the concentration of anionic electrons correspondingly increases (decreases). This leads to opposite changes in the intensities of the edge exciton and *F*-center band.

Figure 2 illustrates the evolution of optical absorption spectra in the nano-physicochemical system *β*-AgI/Mn at a film thickness ratio of *L*_M_/*L*_Hal_ ~ 0.1. Upon completion of the formation and relaxation of the electride phase in the *β*-AgI/Mn system (300 K), the spectrum undergoes a fundamental transformation. Instead of the AgI edge exciton peak, two new absorption peaks, E_1_ and E_2_, emerge in the spectrum. In a magnetic field of 0.85 T, the E_1_ exciton splits into two components. The phase characterized by these two excitons (E_1_ and E_2_) remains stable in an open atmosphere.

The behavior of the AgI/Mn system naturally differs from that of AgI/RE (rare earth element) systems. For active trivalent lanthanides, the driving force for charge transfer is very large (Δ*φ* ≈ +1.94 eV), which ensures the exclusive formation of a homogeneous non-stoichiometric electric berthollide phase without phase separation. In contrast, for divalent manganese, the driving force for charge transfer is small: Δ*φ* = 7.58 − 7.43 = +0.15 eV. As a consequence, the nonlinear open AgI/Mn system delicately balances between two macroscopic attractors, sequentially activated as the concentration of the implanted guest increases:

Attractor 1 (moderate concentration regime): The system forms a continuous series of homogeneous non-stoichiometric solid solutions of variable composition (berthollides) with a high density of interstitial electrons.

Attractor 2 (Mn saturation regime): The limit of non-stoichiometric accommodation of manganese by the AgI matrix is reached. The open system undergoes local bifurcation and converges to the globally most thermodynamically favorable attractor—the stable stoichiometric ternary compound Ag_2_ MnI_4_. At this point, the excess electrons localize in the fixed chemical bonds of the new lattice, and the E_1_/E_2_ excitons mark its successful structural rearrangement. To preserve local electroneutrality, excess silver is coherently displaced through the fast ion transport channels to the film surface, where it crystallizes as dendrites (Figure 8 in [21]).

Thus, the distinct states of the AgI/Mn system do not constitute a logical contradiction but rather successive trajectories of a multistable system in phase space.

The right panel of Figure 2 shows that the E_1_ exciton peak splits into two well-resolved components at 3.328 and 3.372 ± 0.005 eV under a magnetic field *B* = 0.85 T at 293 K. The giant Zeeman splitting of the E_1_ exciton in Ag_2_MnI_4_, reported here for the first time, was measured in 2003 but remained unpublished. At that time, our research focus shifted toward the systematic development of nanoionics of advanced superionic conductors—a direction reviewed in [24], with α-RbAg_4_I_5_ as its paradigmatic representative. This rapidly expanding field took priority, and the Ag_2_MnI_4_ results remained unpublished for an extended period. The energy separation Δ*E* ≈ 44 meV corresponds to an effective exciton *g*-factor of ∼890—approximately three orders of magnitude larger than in conventional semiconductors (*g* ∼ 1–2) and well above the values reported for diluted magnetic semiconductors such as CdMnTe. Notably, this splitting (Δ*E* ≈ 1.8 *k*_B_ *T*) remains clearly resolved at room temperature, a regime where Zeeman splittings of conventional excitonic systems are typically thermally washed out. The splitting is strictly reversible: it appears and disappears upon switching the magnetic field on and off without hysteresis, indicating a paramagnetic rather than ferromagnetic origin.

The excerpts below are taken from the paper *INSERTION OF TRANSITION, RARE EARTH, AND ACTINOID ELEMENTS INTO AgI AND CuI* [21]. This pioneering study investigated nano-systems with high *F*-center concentrations and the formation of electride phases—a term that had not yet entered common usage at the time of publication.

*“...Compounds with the M_2_’M’’ I_4_ general formula containing the nonmagnetic M’—(Ag or Cu) and M’’—(Zn, Cd, Sn, Hg or Pb) elements are presented in the literature. It is solid electrolytes in which under a some raising of temperature (above RT) occur the phase transitions to a superionic state [12,13,14]. Thus, we drawn a conclusion about syntheses of a new member of this family with the Mn^2+^ magnetic actively ions, i.e., Ag_2_MnI_4._ The obtained Ag_2_MnI_4_ films do not decompose on the air. It is a surprise. For example, the meteoric water destroys the Ag_2_ZnI_4_ films [14]. Moreover, the MnI_2_ compound, possible component of Ag_2_MnI_4_, is highly hydroscopic substance. And on the air presence of trace of such substance will instantly break down synthesized film. It means that the way of chemical reaction passes the stage of synthesis of MnI_2_. It is interesting also note, the optical spectra of Ag_2_CdI_4_ contain the E_1_’ and E_2_’’ absorption bands at 2.94 eV and 3.26 eV (300 K), i.e., the spectra of Ag_2_MnI_4_ compound with the E_1_ and E_2_ bands at 3.35 eV and 3.62 eV (300 K) are similar to the spectra of Ag_2_CdI_4_ compound. According [12], the E_2_’’ absorption band at 3.26 eV may be attributed to transitions between excitonic levels localized near 4d (Ag), 5p(I) valence and 5s (Ag) conduction bands of Ag_2_CdI_4_. The E_1_’’ absorption band at 2.94 eV was attributed to 4d^10^ ⟶ 4d^9^ 5s electronic transitions in tetrahedrally coordinated Ag^+^. These transitions are forbidden by the Δl = ± 1 selection rule, but become partially allowed due to the mixing of the 4d states of Ag^+^ with 5p states of iodine...”.*

*“...The question about first elementary chemical acts which take place at an initial stage of insertion of M into Ag- or Cu-iodides with the structure of wurtzite (zincblende) is considered below. The possibility of insertion of a concrete element is determined by many characteristics related to metal and 1b-halide. Among them the first ionization energy of M atom (φ_M_) should have key value. If the first ionization of M occurs the chemically active particles are appearing and a number of kinetic restrictions for insertion are disappearing. Above mentioned that the phase of metal silver was formed in the AgI-Mn system. It may correspond to the reactions: Mn^0^ ⟶ Mn^+^ + e (φ_Mn_ = 717.4 kJ/mol [15]) and Ag^+^ + e^−^ > Ag^0^ (φ_Ag_ = 731.0 kJ/mol). Inequality of φ_Mn_ < φ_Ag_ may be necessary condition for insertion of Mn into silver halides. If so, the manganese will be inserted also into CuI (φ_Cu_ = 745.4 kJ/mol) but iron must not be inserted into both AgI and CuI (φ_Fe_ = 759.3 kJ/mol)...”.*

*“Conclusion. We suppose that the obtained results demonstrate the adequacy of representations (see [3], the section Development of concepts) about a possibility of synthesizing in the Ag(Cu)Hal-M and superionic conductor on the basis of Ag(Cu)Hal-M and systems of a great number (hundreds) of new compounds and materials with high concentrations of optically and magneto active elements. The data and results about synthesis of eight novel compounds were presented in this work. It is necessary to note that the MHal_2_ and MHal_3_ halides of transition (3d, 4d) rare earth (4f) and actinide (5f) elements are highly hygroscopic substances [18]. The way of synthesizing of new compounds (which was proposed in [3] and used in this work) allows to obtain new compounds by passing stage of synthesizing of the mentioned hygroscopic substances. Therefore, new film materials without of traces of hygroscopic phases will be steady against of an atmospheric moisture and able to satisfy to the various applications” [21].*

The excerpts record the following main results:

(1) The successful synthesis of a new magneto-active compound, Ag_2_MnI_4_. Notably, this material demonstrates high chemical stability and does not undergo decomposition via the reverse reaction Ag_2_MnI_4_^−^ > 2AgI + MnI_2_. Consequently, the proposed synthetic route completely eliminates traces of the hygroscopic precursor (MnI_2_) in the final product.
(2) The discovery of a thermodynamic descriptor of the form *φ*_Mn_ < *φ*_Ag_, which establishes a necessary condition for cation exchange and *F*-center formation in nano-physicochemical systems within the framework of the Ag_1−3*x*_M*_x_*I_1−3*x*_ non-stoichiometric model.
(3) The prediction of thousands of new compounds containing optically and magnetically active chemical elements synthesized via routes that bypass the use of hygroscopic di- and trivalent metal halide precursors.

**Figure 3:**
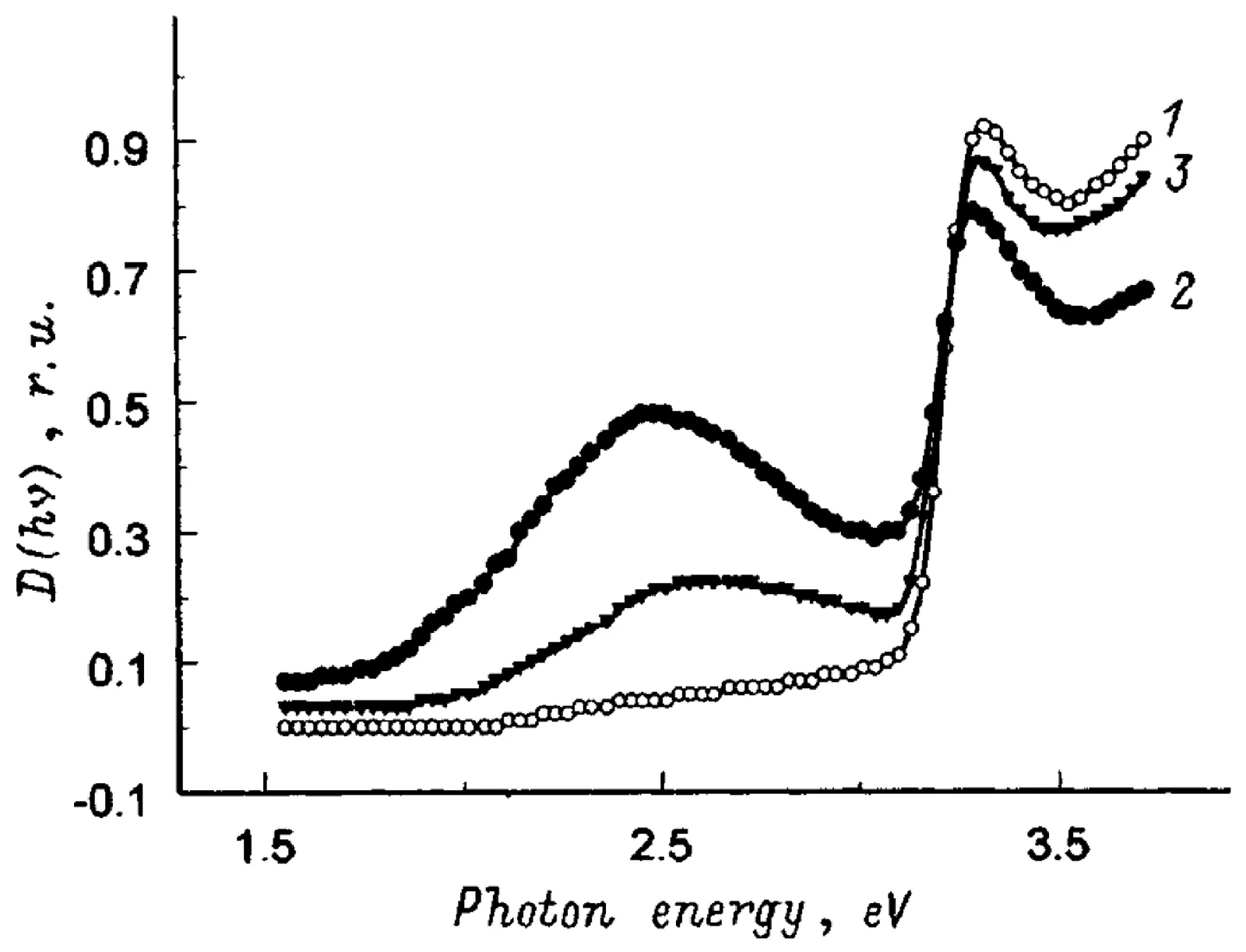
The evolution of the optical density *D*(*hν*) spectra for the electride phase formed in a 350 K α-RbAg_4_I_5_/Yb thin film. 1—initial 160 nm superionic conductor matrix α-RbAg_4_I_5_, 2—sample after deposition of a 4 nm Yb layer; 3—stabilized state after 43 h of dry air storage [19]. The comparison between traces (1) and (3) highlights the long-term stability of the thin film system following vacuum processing [19].

Figure 3 illustrates the evolution of optical absorption spectra at low concentrations of interstitial electrons in the halide matrix, exemplified be the α-RbAg_4_I_5_/Yb system with a film thickness ratio of *L*_M_/*L*_hal_ ~ 0.01. The optical response of the electride phase is characterized by the concurrent quenching of the edge exciton peak and an increase in absorption within a broad spectral band corresponding to the bandgap energies. This phenomenon indicates the filling of iodine vacancies with interstitial electrons (electride formation): the population of anionic sites available for exciton generation decreases as they are occupied by denucleated electrons. The edge exciton peak acts as a sensitive indicator of the electride phase state [11,12,13,14]. Its quenching reflects the physical process wherein ionic bonds are superseded by localized interstitial electrons, stemming directly from the halogen deficit in regular anionic states.

The concentration of electron centers within the bandgap of the ionic conductor and its correlation with the number of departing halogen anions are quantitatively described by Smakula’s formula. Originally proposed in 1930 for the spectroscopic determination of isolated *F*-center concentrations in alkali halide crystals, this relationship is expressed as

(2) $$N_{F}=\frac{1}{f}\cdot \left[\frac{9n}{(n^{2}+2{)}^{2}}\right]\frac{m^{*}c}{\pi e^{2}}\alpha_{\max}W$$

where *f* is the oscillator strength of the optical transition, *n* is the refractive index of the ionic conductor (*n* ≈ 2), m^*^ is the electron effective mass at the bottom of the conduction band (m^*^ ≈ 0.25m_0_), α_max_ is the optical absorption coefficient at the maximum of the *F*-center band, and *W* is the full width at half maximum (FWHM) of the *F*-band. For the modified α-RbAg_4_I_5_/Yb system, we experimentally observed a broad absorption band with a maximum at photon energy of 2.4 eV and a FWHM of 0.4 eV. According to Equation (2), this feature corresponds to an interstitial electron concentration of *N*_F_ ~ 3·10^20^ cm^−3^ (see Figure 3). This concentration corresponds to 1.5–1.8 guest Yb^3+^ ions per 100 mobile cations Ag^+^. In the resulting electride phase, anionic electrons are localized within approximately every second cubic unit cell of the parent advanced superionic conductor RbAg_4_I_5_.

In the optical density spectra, the number of anion positions occupied by denucleated electrons correlates directly with the quenching of the edge exciton intensity. This spectral redistribution obeys the oscillator strength sum rule and can be described by the optical balance equation:

(3) $$\frac{d}{\mathrm{dy}}\left[\int \alpha_{\mathrm{ex}}\left(\nu \right)\mathrm{d\nu}\right]\propto -\frac{d}{\mathrm{dy}}\left[\int \alpha_{F}\left(\nu \right)\mathrm{d\nu}\right]$$

**Figure 4:**
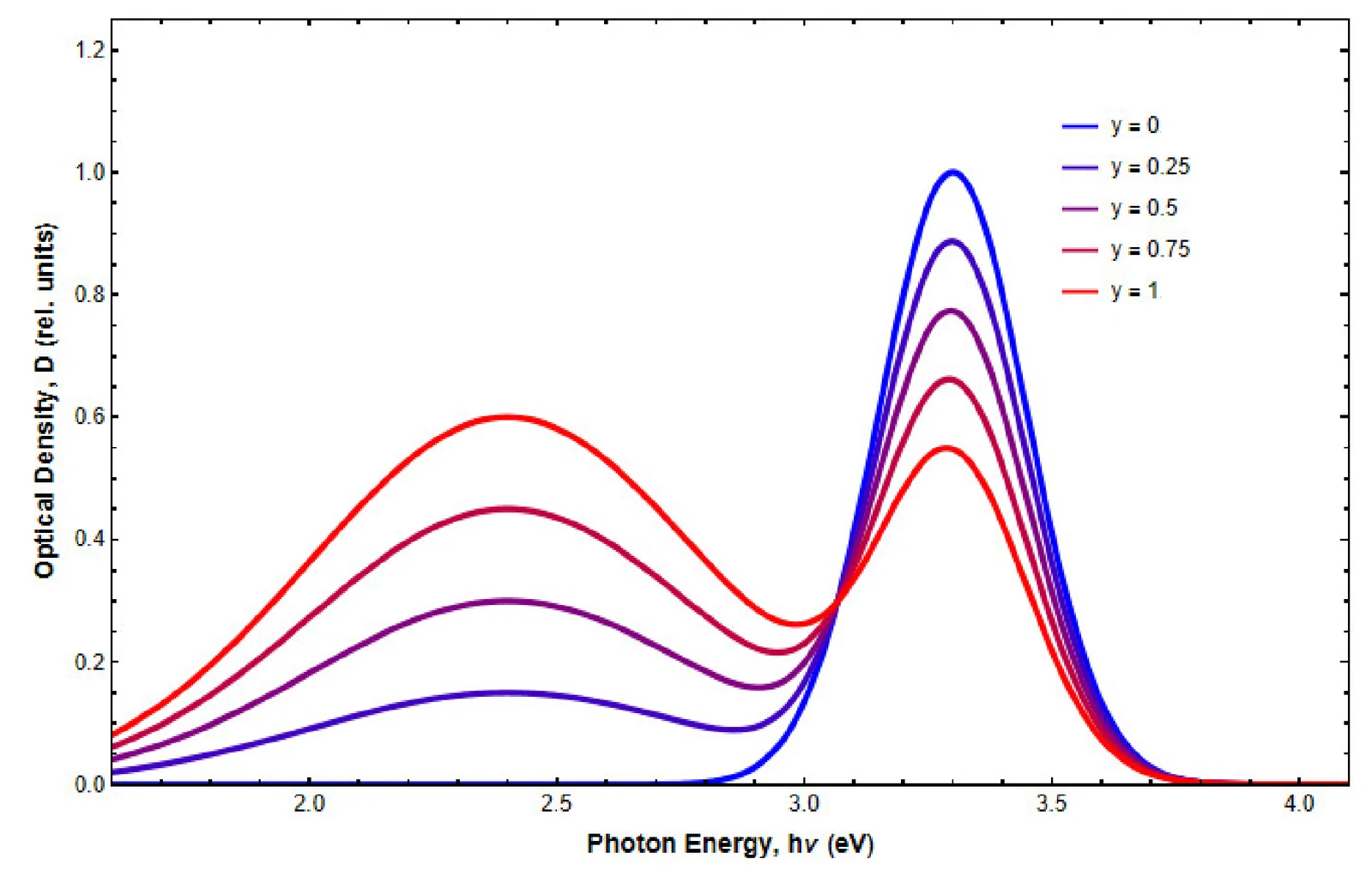
Numerical analysis of the oscillator strength transfer between the edge exciton (3.3 eV) and the *F*-center band (2.4 eV) in α-RbAg_4_I_5_/Yb systems within thin films (80–200 nm) of electride phases. The fundamental continuous absorption region lies outside of the scope of the current defect chemistry simulation. Conditional concentrations *y* of a dopant in α-RbAg_4_I_5_: 0, 0.25, 0.5, 0.75 and 1.

Figure 4 presents the simulated optical absorption spectra based on the experimentally observed evolution of *D* in thin films (80–200 nm) of the superionic conductor RbAg_4_I_5_; see Figure 3. As the interstitial electron concentration increases, the edge exciton peak quenches at photon energy of 3.3 eV, while the broad absorption band with a maximum at 2.4 eV and *W* ≈ 0.4 eV grows in intensity proportionally.

The spectral evolution indicates an inverse linear relationship between the integrated absorption areas of the edge exciton and the interstitial electron band. Equation (3) does not capture the critical observation that at *N*_F_ ~ 3·10^20^ cm^−3^ the blue-shift of the maximum upon decreasing the dopant concentration (*y*) signifies a weakening of the interaction between localized electrons. Notably, the absorption spectra show no significant variation across a broad range of systems incorporating trivalent guest ions. The independence of the spectral profile from the atomic number confirms that the optical response is determined by interstitial electrons rather than local electronic transitions within the guest ions.

The analysis of the properties of ionic conductor matrices with high anionic electron concentrations points to physical limitations regarding the applicability of the classic Smakula approximation. Calculations indicate that expression (2) underestimates the concentration values when *N* > 10^18^ cm^−3^. This deviation stems from the onset of electronic delocalization as the inter-center distance decreases and the electron wave functions overlap. Consequently, many-body interactions redistribute the total spectral weight, suppressing the individual dipole strength *f*_eff_ of each localized *F*-center below its nominal single-defect value. The systematic deviation at high concentrations necessitates an empirical modification of Smakula’s equation [25]. We therefore introduce a scaling coefficient *Γ*(*N*) into expression (2) as a metrological framework:

(4) $$N_{F}=\Gamma \left(N\right)\frac{1}{f_{0}}\cdot \left[\frac{9n}{(n^{2}+2{)}^{2}}\right]\frac{m^{*}c}{\pi e^{2}}\alpha_{\max}W$$

where *f*_0_ is the standard oscillator strength for *F*-centers at low concentrations. The factor *Γ*(*N*) has the form

(5) $$\Gamma =1+\frac{\gamma \cdot (N/N_{c})}{1+\delta \cdot (N/N_{c})}$$

where *n_c_* ∼ 10^20^–10^21^ cm^−3^ is the Mott density determined by the onset of wave function overlap in the ionic host, consistent with the Mott criterion *a_B_*^∗^·*n_c_*^1/3^ ≈ 0.25 using the effective Bohr radius *a_B_*^∗^ = *a_B_*·*ε*/(*m*^∗^/*me*) with the ionic dielectric constant of the superionic conductor. The parameters *γ* and *δ* carry physical meanings: *γ* ≈ 0.15–0.30 characterizes the initial rate (slope) of oscillator strength degradation, while *δ* ≈ 0.05–0.15 sets the limit for complete Mott-type broadening of the wave functions. Both parameters are intended to be experimentally calibrated in future UHV studies through systematic comparison with independent film thickness measurements, with the expected range *Γ*_max_ ≈ 2–4.

The calculated order-of-magnitude values of electron concentrations exceeding 10^21^ cm^−3^ were established through a comprehensive, independent, two-stage methodology based on direct gravimetric and geometric comparison of thickness balances and molar masses for co-deposited layers. In the first stage (*N_F_* ≤ 10^20^ cm^−3^), the optical density data were correlated with the thickness ratio of the matrix film and the deposited metal layer. In the second stage (*N_F_* > 3·10^20^ cm^−3^), the density was estimated from the thickness ratio of the films.

1. Low-concentration regime (≤3·10^20^ cm^−3^) and validation of the Smakula formula: At the initial ultra-thin limits of co-deposition (thickness ratio of the guest metal film to the host matrix on the order of 1:100), the concentration values derived from the absolute film thickness ratio are in close agreement with those calculated using the classical linear form of the Smakula equation. This agreement experimentally establishes the unexpected applicability of the linear Smakula approximation up to record densities of the order of 10^20^ cm^−3^. In this regime, the formula serves as an adequate metrological probe for estimating the strength of electron–anion interactions and for finely tracking the onset of oscillator strength degradation.

2. Extreme-concentration regime (>10^21^ cm^−3^) and the metrological status of *Γ*(*N*): As the co-deposition parameters shift into the macro-alloying range (film thickness ratio approaching 1:10), the isolated defect assumption breaks down. Individual electronic wave functions begin to undergo strong spatial overlap, violating the premise of non-interacting centers. This leads to a nonlinear attenuation of oscillator strengths, which is independently confirmed by optical transmission data and X-ray diffractograms recording a distinct structural phase transition.

The correction function *Γ*(*N*) with a phenomenological parameter *γ* ≈ 0.15–0.3 is presented as a predictive formulation developed to account mathematically for this nonlinear attenuation effect in regimes where the classical approximation ceases to hold. This formulation is introduced strictly as a prospective theoretical framework for future experimental studies rather than an established empirical calibration or an a posteriori fitting to manipulate the existing historical figures retroactively. By systematically comparing the shifting integrated optical absorption areas with absolute data from a quartz crystal thickness–mass monitor, future researchers will be able to calibrate *Γ*(*N*) for different families of ionic conductors.

Regarding the uncertainty, we emphasize that these calculated values represent order-of-magnitude approximations, which are natural for highly concentrated quantum-confined systems where the carrier concentration exceeds the Mott delocalization threshold.

The spectra in Figure 1 for the AgI/Nd system capture the attainment of critical anion electron concentrations, which triggers a transition into a qualitatively new electride state. Experimental validation of this structural shift is supported by X-ray diffraction data from the analogous AgI/Sm system, where a structural transformation of the dynamically forming framework was observed at a component ratio of *n_Sm_*/*n*_Ag_ ~ 0.1. The corresponding diffractograms reveal a pronounced lattice expansion, with characteristic d-spacing reaching 0.99 nm. Within our interpretative model, this structural feature signifies an approximate doubling of the unit cell parameter relative to pristine silver iodide, serving as primary structural evidence consistent with the formation of a new ordered electride phase.

Thus, the collected experimental data demonstrate that in ionic conductor matrices possessing excess positions for mobile-type ions, electron–anion densities exceeding 10^21^ cm^−3^ can be achieved. We infer that the very presence of these excess positions in the *H*-matrix, which sustain ionic motion, serves as the second necessary condition for initiating reactivity in the systems under study.

These findings pave the way for broadening the family of compounds capable of hosting dynamically forming electrides. The formation of these phases represents a self-organizing process governed by the Δ*φ*_HG_ > 0 descriptor and enabled by the presence of excess cationic vacancies within the *H*-lattice.

This unique combination of high anion–electron density and ionic conductivity renders these materials promising candidates for a diverse range of technologies. Increasing the concentration of anionic electrons in matrices drives a transition from localized *F*-center states to delocalized Fermi liquid behavior: at low concentrations, excess electrons occupy isolated anion vacancies as insulating species; increasing the density of such electrons leads to greater wave function overlap and impurity band formation, reaching critical densities exceeding 10^21^ cm^−3^ for metallic conduction via interstitial bands; and finally, further increases in density can destabilize the lattice due to strong Coulomb repulsion between denucleated electrons. Therefore, electride materials can be used as follows.

- In fundamental and applied spintronics: The co-injection of 3d-, 4f и 5f elements into labile host matrices enables controlled electron spin manipulation via the formation of local magnetic moments and exchange interactions within non-stoichiometric phases of variable composition.
- In fundamental condensed matter physics: Investigation of strongly correlated electron systems, Mott transitions, and heavy-fermion phenomena enabled by tunable carrier density.
- In heterogeneous catalysis: The high reducing capacity of interstitial electrons enables these electride phases to serve as sustainable catalyst materials.
- In emission electronics: The low work function of interstitial electrons enables efficient field emission, including switchable dual-mode sources capable of generating electron and ion beams along the same axis.
- Biomedicine and antimicrobial applications (prospective): Silver iodide is a proven biocidal material used in antimicrobial compositions, and its nanostructures exhibit broad-spectrum antibacterial activity through Ag^+^ release and ROS (reactive oxygen species) generation. The electride phase demonstrated in an AgI-based matrix may introduce a complementary biocidal mechanism: direct electron transfer to bacterial membranes via the established extracellular electron transfer pathway, disrupting transmembrane potential. The extension to biocompatible organic Ag^+^-conducting matrices is supported by the matrix-independent nature of the Δ*φ*_HG_ descriptor and the inherently high structural lability of polymer matrices. These directions are identified as scientifically motivated prospects for future experimental investigation in antimicrobial applications.

## 3. Discussion of Experimental Results

### 3.1. Properties of Solid-State Ionic Conductors and Host Structure Lability as Criteria for Electride Material Screening

The criterion *φ*_MH_ − *φ*_MG_ = Δ*φ*_HG_ > 0 (Equation (1)) determines the feasibility of non-stoichiometric compound formation in host–guest systems of type *H*-matrix/M_G_. This descriptor defines the fundamental capability of a physicochemical system to form electride phases. However, achieving high concentrations of interstitial electrons requires that two additional criteria be satisfied. First, the matrix must possess a high density of crystallographic or interstitials positions suitable for accommodating *M*_G_^+^ ions. Second, the matrix must be capable of continuously adapting its structure upon changes in the concentrations of both *M*_G_^+^ -ions and anionic electrons.

In the case of solid-state ionic conductors, the first additional condition is met automatically: the number of vacant crystallographic and interstitial positions is several times greater than the number of mobile-type ions, even in poorer ionic conductors where transport occurs via an interstitial mechanism (i.e., through spaces between the main lattice sites along the so-called interstitial positions). These positions ensure the possibility of barrier-free insertion of guest *G*-atoms into the host *H*-matrix and the subsequent transport of *M*_G_^+^ ions within it. In studies [19,20,21,22,23,24], α-RbAg_4_I_5_, α-CsAg_4_Br_3−x_I_2+x_, and AgI и CuI halides were utilized as the *H*-matrix.

In superionics, the number of accessible positions exceeds the number of the mobile Ag^+^-ions themselves by several times [26]. Among these, a distinct class of advanced superionic conductors is identified, representing solid electrolytes with the highest ion transport properties: an ionic conductivity of *σ*_i_ > 0.1 Ohm^−1^ cm^−1^ at 300 K and an ion transport activation energy of *E*_a_ ≈ 0.1 eV [24]. Such compounds, for instance, α-AgI and α-RbAg_4_I_5_, exhibit cubic symmetry and possess a unique structure. This structure consists of a rigid, close-packed anionic sublattice, wherein mobile ions are distributed over tetrahedral positions and move through fast ionic transport (FIT) channels. The FIT channels are composed of face-sharing tetrahedra and form a three-dimensional (3D) network at the unit cell level [24,27,28].

The issue of satisfying the second additional condition requires special consideration. The physical situation in the field of searching and material design for inorganic electrides stable under ambient conditions requires careful analysis of the elastic basis of condensed media. The traditional macroscopic triad of Born–Huang elastic stability, based on the components of the elasticity tensor, operates within the mechanics of continuous media and describes the response to homogeneous external macro-loads [29]. The Born–Huang criteria are widely used in materials science to predict the mechanical properties of materials, analyze their stability, and screen promising compounds for practical applications. In the context of electrides, however, these macroscopic parameters lose their predictive power. Upon localization, an interstitial electron interacts not with an abstract elastic continuum, but with the nearest-neighbor *H*-matrix cations defining the void walls. Within this nanoscale region, phase stability is governed by the lattice’s capacity for microscopic electrostatic screening and local void relaxation.

In solid-state physics, the elastic stability of crystal lattices is evaluated using criteria defined via the elastic constants (elastic moduli) of the crystal. These criteria are based on the fundamental requirement that the elastic energy of the system must be positive. To ensure the mechanical stability at a fixed temperature *T*, all principal minors (determinants) of the elastic modulus matrix in Voigt notation must be strictly positive. Satisfaction of these inequalities guarantees the mechanical stability of the lattice against arbitrary infinitesimal perturbations. The specific form of these conditions depends entirely on the crystal symmetry.

For cubic crystals, the stability conditions take the following form:

*C*_11_ > |*C*_12_|; *C*_11_ > 0; *C*_44_ > 0; *C*_11_ + 2*C*_12_ > 0. For crystals belonging to other crystal systems, the conditions become significantly more complex and involve multi-component combinations of the corresponding elastic constants *C*_ij_. Crucially, satisfying the Born criteria guarantees mechanical stability only in the infinitesimal limit.

In a number of cases, in addition to satisfying the Born criteria, it is necessary to calculate the normalized Cauchy pressure. This quantity serves as a robust descriptor of local microscopic lability and relaxation of voids. The Cauchy pressure, normalized to the bulk modulus (*B*), takes the following form:

*П*_Lab_ = (*C*_12_ − *C*_44_)/*B* (6)

Importantly, in cubic crystals, the difference between the elastic moduli (*C*_12_ − *C*_44_) reflects the degree of deviation from the classic Cauchy relation, which postulates the equality of these constants for strictly central Coulombic bonding forces.

A negative value of *П*_Lab_ indicates the dominance of rigid directional covalent or hyperelastic forces that block the deformational response of the lattice voids. Corresponding values can be obtained, for instance, for MgO (−0.373) and LiF (−0.229) [30,31]. The condition *П*_Lab_ ≈ 0 determines an ideal balance of the central interatomic potentials in rigid non-conducting alkali halide crystals, such as NaCl, KCl. Conversely, high positive values *П*_Lab_ signal shear mode softening, an increasing contribution of non-central interatomic interaction forces, and increased elastic lability of the *H*-matrix framework.

This trend is observed when transitioning from rigid dielectrics to solid-state ionic conductors and superionics. The latter possess the band structure of ordinary dielectrics and are characterized by a highly polarizable *H*-matrix. Possessing filled d-electron shells, these cations give rise to values of *П*_Lab_ that are high for the cubic crystal system. For several compounds, experimentally determined elastic moduli can be found in the literature. Calculations for *П*_Lab_ indicate increasing lability: *П*_Lab_ rises from the moderately labile InSb (0.135) and fluorite CaF_2_ (0.129) [32,33] to the superionic rubidium electrolyte RbAg_4_I_5_ (0.379) [34] and the copper halides CuCl (0.577) and CuBr (0.553) [35], reaching a maximum for the cubic modifications of the silver halides AgCl (0.685), AgBr (0.626), and AgI (0.614) (sphalerite structure) [36,37]. The abundance of vacant crystallographic or interstitial sites in superionics, combined with strong polarization potential, provides efficient accommodation for high concentrations of interstitial electrons and guest ions.

Finally, we would like to conclude with a broader epistemological reflection. The entire historical paradigm of color centers was profoundly shaped by the materials chosen by the legendary Göttingen school of R. Pohl. In the 1930s and 1940s, Pohl and his contemporaries conducted their pioneering experiments on rigid, non-conducting alkali–halide crystals (such as KBr or NaCl), where central forces dominate and the elastic lability is negative (*Π*_Lab_ < 0). In such crystals, anionic electrons could only form isolated point defects.

Had the Pohl school employed labile structures with fast ion transport channels for their additive coloration experiments, they could well have encountered the full spectrum of phenomena associated with high concentrations of anionic electrons, and the foundations of inorganic electride science might have been laid decades earlier. The present work seeks to move beyond this historical path dependence by extending solid-state physics into the domain of dynamically formed electrides. Thus, in the search for electride phases dynamically forming within nano-physicochemical systems based on solid-state ionic conductors, the following screening criteria have been established:

- Thermodynamic descriptor (1): Strict fulfillment of the condition Δ*ϕ*_HG_ > 0, which governs the fundamental reactivity of the system and enables the barrier-free transitions of electrons to anion vacancies.
- Structural redundancy of voids: A high density of vacant crystallographic and (or) interstitials positions within the *H*-matrix that significantly exceed the concentration of mobile primary ions, thereby providing fast transport channels.
- Elastic lability of the framework: A shift of the normalized Cauchy ratio to large positive values *П*_Lab_ = (*C*_12_ − *C*_44_)/*B* > 0.35, serving as a direct indicator that shear mode softening and the lattice deformability relate to microscopic Coulombic compression of the void walls. An important objective is to establish the relationship between the lower bound *П*_Lab_ and the maximum achievable anion–electron concentration. Solving this task is essential for designing electride phases with a concentration of electron–anions exceeding 10^21^ cm^−3^. To the best of our knowledge, such a task has not been previously formulated in the literature.

As demonstrated by our experimental results, the screening criteria presented above are satisfied for all investigated physical–chemical *H*/*G* thin-film systems based on solid-state ionic conductors. Consequently, these criteria can serve as reliable descriptors for material selection in the search for electrides.

### 3.2. Thermodynamic Principles and Reaction Mechanisms in the Formation of Electrides Within Open Nanochemical Systems

The experimental data and results presented above characterize chemical interactions occurring at atomically clean interfaces within nano-physicochemical systems based on solid-state ionic conductors under ultrahigh-vacuum conditions. The initial objective of our study was to lay the foundations for nanoionics—a discipline first termed and conceptualized in [38]. Exploratory studies on rare-earth insertion into solid electrolyte films revealed radical spectral transformations during *F*-center formation and evolution within nano-systems. The research project [39] culminated in the discovery of dynamically forming electride phases in solid-state ionic conductor systems: nanoionics became directly linked to the predictive screening, design, and synthesis of electrides—a field of materials science that has advanced rapidly since the 2000s. Furthermore, other insights from nanoionics are already actively informing current electride research ([40,41]).

What initially appears as a fundamental contradiction between theoretical predictions and experimental reality in open nano-physicochemical systems is revealed to be a resolvable paradox upon recognizing the distinct physicochemical behavior of matter at the sub-nanometer scale. Let us consider this subject in detail.

Standard thermodynamic calculations for the AgI + Al system under vacuum (precluding oxide formation) predict a classical displacement reaction in which aluminum reduces silver:

3AgI + Al ⟶ AlI_3_ + 3Ag↓ (7)

The change in Gibbs free energy, Δ*G*, for the macroscopic pathway (7) at 300 K is Δ*G*^0^ ≈ −102 kJ/mol, accompanied by a significant exothermic effect, Δ*H*^0^ ≈ −117 kJ/mol. This heterogeneous interaction is described in [42], while the specific characteristics of the Al/AgI interface coupling at the nanoscale are studied in [43]. General reference data on the metal halide reduction are summarized in monograph [44]. No data on alternative chemical reactions within the AgI/Al system are currently available in the literature. Nevertheless, in situ experiments [19,20,21,22,23,24] refute the displacement reaction of metallic Ag (7), despite closely comparable macroscopic thermodynamic parameters for the AgI/RE and AgI/Al systems. In both cases, the driving force behind the nanoscale interface self-organization is governed by the criterion *φ*_MH_ − *φ*_MG_ = Δ*φ*_HG_ > 0. For the Ag-Sm (1.93 eV) and Ag-Al (1.59 eV) pairs, this effective difference in first ionization potentials varies by less than 18%.

Notably, no visible deterioration—such as opacification—was observed in transparent thin-film samples after storage in air for hours, days, or even weeks. This applied to RbAg_4_I_5_–Sm, AgI–Al, and AgI–rare-earth (La, Ce, Nd, Sm, Tb, Tm, Yb, Lu, Sc, Y) systems.

This stability is noteworthy, because the expected displacement products would be highly hygroscopic. Aluminum iodide instantly absorbs moisture from air, undergoes partial hydrolysis, converts to a basic salt, and releases gaseous hydrogen iodide. Anhydrous lanthanide iodides (LnI_3_) likewise show extreme affinity for water: they instantly absorb moisture from air, undergo deliquescence (dissolving in their own absorbed water), and hydrolyze with release of HI/I_2_.

It is reasonable to assume that at the nanoscale heterointerface, the classical chemical displacement pathway is suppressed, presumably due to a high nucleation barrier associated with bulk phase growth under spatial confinement. In contrast, a thermodynamically driven self-organization occurs within labile *H*-matrices, resulting in the formation of non-stoichiometric electride phases. The driving force arises from three contributions: exothermic charge transfers due to the potential difference of the *H*/*G* pairs, the Coulombic solvation of electrons in sub-nanometer voids, and minimal elastic energy required for the structural relaxation of the labile framework (*П*_Lab_ > 0.35). Consequently, within the interpretative framework of interface-driven self-organization, the proposed thin-film configuration is suggested to represent a thermodynamically favorable alternative to the bulk macroscopic silver displacement reaction.

Thus, the apparent paradox is resolved by recognizing that the primary product of the nanoscale interaction in AgI/Metal-type heterosystems under ultrahigh vacuum conditions [19,20,21,22,23,24] is a non-stoichiometric halide of variable ([M_x_Ag_1−3x_I_1−3x_]^3x+^·(e^−^)_3x_) composition, which represents a new class of thermodynamically stable electrides–berthollides (stable in an inert atmosphere at 1 atm and 300 K).

In the classical non-stoichiometric halides studied by the Göttingen school (R. Pohl), the bulk concentration of *F*-centers was strictly limited by a thermodynamic threshold on the order of 10^17^ cm^−3^, corresponding to dilute point defects physics in rigid alkali halide matrices. When this threshold was exceeded, the *F*-centers begin to coagulate into colloidal metal particles. In such crystals, the normalized Cauchy pressure *П*_Lab_ = (*C*_12_ − *C*_44_)/*B*, approaches zero, as *C*_12_ − *C*_44_ ≈ 0.

The dimensionless descriptor *П*_Lab_ allows for quantitative assessment of the host crystal structure’s lability with respect to local internal influences, including nanoscale adaptation and the “Coulombic compression” of voids around the incorporated guest ions and interstitial electrons. Specifically, for the low-temperature cubic modification of AgI (sphalerite structure, γ-AgI), evaluation based on experimental elastic constants yields a high positive value of lability descriptor, *П*_Lab_ = 0.614. This value is derived from the crystallographic transformation of the wurtzite elastic moduli. For the advanced solid electrolyte RbAg_4_I_5_, this parameter computed from the elastic moduli also exhibits an anomalously high value of *П*_Lab_ = 0.379. Such data of the Cauchy descriptor directly indicate the non-central character of interatomic interactions within silver halide lattice, which ensures high matrix lability toward local structural relaxation.

In the first approximation, the nanoscale formation of the non-stoichiometric electride phase based on silver iodide is described by the following material balance equation:

(8) $$\mathrm{AgI}+xM^{0}\overset{\mathrm{self}-\mathrm{organization interface}}{\to }{\left[M_{x}Ag_{1-3x}I_{1-3x}\right]}^{3x+}\cdot {\left(e^{-}\right)}_{3x}+3x\mathrm{AgI}↓$$

The presence of iodine vacancies is essential to enable a barrierless incorporation process. Here, the notation (e^−^)_3x_ denotes the valence electrons of the *G*-metal (e.g., Sm or Al) that become localized within the lattice voids. These interstitial electrons occupy vacant regular anionic sites of the halide framework. According to the Frenkel model, point defects are spontaneously generated at any *T* > 0 K. Due to dissipative cooperative mass transport, an excess of silver and iodine ions is coherently displaced from the bulk toward the surface, where they form a stable secondary phase sink 3xAgI.

The results of in situ spectral and X-ray diffraction measurements [19,20,21,22,23,24] clearly demonstrate that the synthesized products of nanoscale interaction are completely inert to atmospheric moisture vapors and exhibit no macroscopic signs of hygroscopicity. Within our scenario, this phenomenal stability is suggested to indicate the suppression of alternative hygroscopic lanthanide salt phases (such as LnI_3_ individual phases).

According to standard handbooks, the absolute majority of individual trivalent and divalent halides of lanthanides and transition metals are extremely hygroscopic, highly deliquescent in air, and prone to rapid hydrolysis. Individual aluminum triiodide (AlI_3_), is a thermodynamically mandatory product of the classical macroscopic displacement reaction (7), also belongs to these unstable media.

Thus, the noted thermodynamic contradiction is resolved be recognized that classical literature sources [42,43,44] describe reaction (7) under standard macroscopic conditions, whereas in situ experiments [19,20,21,22,23,24] reveal distinct behavior: in nano-systems, the thermodynamic and kinetic control, as well as the selectivity of reaction pathways, differ substantially. The alternative reaction pathway ensures the synthesis of inorganic electrides that remain stable in a humid atmosphere at 300 K.

Direct X-ray diffraction data record a regular decrease in the unit cell parameter of RbAg_4_I_5_, providing experimental evidence of the Coulombic compression of anionic electrons (high lability of the host crystal structure). Based on the spontaneous character of the process, electride formation is suggested to represent a thermodynamically favorable alternative that is accompanied by a qualitative decrease in the total Gibbs free energy of the system. In this description, the ensemble of interstitial electrons must be characterized by its own partial molar potential. The quantitative analytical derivation of the contribution to the total Gibbs energy of the electride phase remains beyond the scope of the present work and constitutes a task for future theoretical studies.

From the standpoint of non-equilibrium thermodynamics, process (8) corresponds to the formation of an electride phase within an open, non-equilibrium system. The dynamically forming electride is stabilized by the dissipative, cooperative mass transport of silver and iodine components from the bulk onto the surface. The question regarding the ratio of the Gibbs free energies for the products of reactions (7) and (8) requires special theoretical consideration.

The proposed interfacial electride transformation pathway is consistently corroborated by the following two independent, self-consistent lines of evidence:

1. Direct experimental demonstration of Δ*G* < 0: The inequality Δ*G* < 0 is directly demonstrated by the spontaneous, activation-free character of the mass and charge transfer process at the interface, which proceeds at room temperature (293 K) inside a high-vacuum chamber during thin-film deposition. According to the Second Law of thermodynamics, since the reaction proceeds without external energy input, the Gibbs free energy of the combined system (the open system together with its immediate surroundings) must decrease (Δ*G* < 0), and the stable existence of the resulting electride phases serves as unambiguous empirical evidence of this thermodynamic driving force.

2. Physical limitations of pathway calculations in superionics: We argue that a minimum-energy static pathway calculation cannot be adequately implemented for these particular systems. The continuous fast-ion-transport channels of our host matrices feature a liquid-like, highly compliant cation sublattice devoid of fixed long-range translational coordination for the intercalated guest species.

Thus, the macroscopic sign Δ*G* < 0, stated in the Abstract, is rigorously justified by the direct experimental spontaneity of the process, while an explicit numerical calculation remains beyond the scope of conventional static models.

### 3.3. Design Strategies for the Use of New Classes of Matrices for Dynamically-Forming Electrides

The AgI-MI compounds (M = Rb, K, and NH_4_) belong to matrics where the topology of migration channels is formed by face-sharing polyhedra representing the interstitial space for cations. Generalizing the concepts [1] regarding the expansion of the chemical-technological space for electride screening, one must transition from rigid lattices to plastic organic-inorganic structures to search for promising latent matrices.

For the synthesis of dynamically forming electrides, superionic conductors in quasi-binary systems with a common anion (e.g., PyrAg_5_I_6_, and Pyr_5_Ag_18_I_23_) can be proposed as promising latent matrices [45,46,47]. The difference between the first ionization potentials of the metals in the *H*-matrix and the guest donor continues to serve as the primary thermodynamic descriptor for electron transfer into solid-state ionic conductor matrices [19,20,21,22,23,24]: *φ*_MH_ − *φ*_MG_ = Δ*φ*_HG_ > 0 (1).

It should be noted that the lability parameter may assume a different analytical form in organic-inorganic ionic conductors serving as matrices for dynamically forming electrides. Upon transitioning to plastic hybrid molecular-ionic frameworks, the expression *П*_Lab_ for the cubic crystal system requires modification. In organic-inorganic conductors, along with the shear compliance of the rigid sublattice, a critical role is played by the rotational (librational) degrees of freedom of the organic cations (e.g., pyridinium), their spatial reorientation, and high local dipolar polarizability. Consequently, the analytical descriptor of microscopic lability must also account for the dielectric susceptibility tensor and the rotational viscosity parameters of the hybrid framework, which govern the Coulombic compression of voids after transfer of electrons.

The transition to super-labile structures will enable record-high concentrations of interstitial electrons. Below, we analyze several experimental studies whose results may open new avenues for the synthesis of electride phases stable at 1 atm.

In pyridinium hexaiodopentaargentate ([C_5_H_5_NH]Ag_5_I_6_), the structural transition to a highly conductive state above 50 °C opens pathways for ionic transport of silver ions. These newly opening face-sharing migration channels can serve as sites for the incorporated guest species during the formation of a stable non-stoichiometric electride [48].

The analysis of general ion transport regularities in solid electrolytes indicates that directional heterovalent doping and the design of amorphous or polymeric matrices enable artificial tuning of high concentrations of interstitial voids and defects within the *H*-matrices. The presence of branched networks of face-sharing polyhedra with minimal steric barriers in narrow cationic windows represents a key crystallochemical requiremet for fast ion kinetics [49].

The synthesis of AgI-based superionic glasses containing monomeric alkylammonium cations (e.g., TMAI and TEAI) demonstrates high ionic conductivity at 300 K [50]. Due to their low surface charge density and strong polarizability, large tetraalkylammonium cations screen Coulombic interatomic interactions and create geometrically redundant, hyperelastic migration channels for the thresholdless electron localization [50].

Incorporating organic alkylammonium cations (e.g., derived from tetramethylammonium iodide, TMAI) into glassy sulfide-based Li-ion conductors ([TMA]I-LiI-Li_2_S-P_2_S_5_) enhances structural lability of the framework [51]. In such systems featuring Li^+^ -conductors, elements with lower first ionization potential of lithium *φ*_1_ = 5.39 eV) are suitable for electride formation: Cs (*φ*_1_ = 3.89 eV), with a potential difference relative to lithium of Δ*φ* ≈ 1.5 eV; Rb (*φ*_1_ = 4.17 eV), K (*φ*_1_ = 4.34 eV), Na (*φ*_1_ = 5.14 eV); and Ba (*φ*_1_ = 5.21 eV).

A materials science analysis of the application of porous metal-organic frameworks (MOFs) as solid electrolytes demonstrates that targeted pore engineering and the presence of highly ordered open metal sites enable the selective encapsulation and isolation of large anions, thereby ensuring high ionic conductivity at 30 °C, which positions them as promising candidates for dynamically forming electrides [52].

The synthesis of a canonical family of double salts stable at room temperature with the general form (Me_4_N)_2_Ag_13_I_15_ indicates the feasibility of achieving high ionic conductivity in electride phases. X-ray diffraction analysis reveals spatial disordering of the mobile ion sublattice and the presence of redundant face-sharing migration channels, which can provide conditions for formation of the electride phase [53].

Studies of thermoelectric power and the correspondence between conductivity activation energy of transport enthalpy in [(CH_3_)_4_N]_2_Ag_13_I_15_ and [(C_2_H_5_)_4_N]_2_Ag_13_I_15_ compounds, which exhibit an ionic conductivity of approximately 0.02–0.04 Ohm^−1^·cm^−1^, confirm of a highly disordered cationic sublattice [45].

A large-scale screening of fifty-eight binary systems based on silver iodide with saturated and unsaturated azacyclic iodides of the general form (1 − x)AgI·xQI demonstrates the achievement of high ionic conductivity (up to 0.065 Ohm^−1^ cm^−1^ at 22 °C). It is critically depending on the molecular weight and molecular volume of the organic cation. The experimentally derived structural threshold for phase stability (exceeding 290 g/mol for QI salts) highlights the feasibility of utilizing bulky heterocyclic pyrrolidinium and piperidinium ions for the targeted expansion of free volume and the generation of geometrically redundant interstitial voids adapted for the thresholdless localization of a denucleated electrons [54].

The synthesis of porous iodoargentate frameworks of variable composition based on cubane-like [Ag_4_I_4_] clusters, formulated as [R_2_Ag_4_I_6_]_n_ (where R is an N-alkyl-4-cyanopyridinium), enables the expansion the free volume and unit cell papameters via targeted elongation of the pyridinium N-alkyl chains. The resulting spatial rearrangement of the anionic sublattice from two-dimensional sheets to open three-dimensional networks with an α-cristobalite topology ensures high channel lability, enabling effectively localizing non-stoichiometric interstitial species [55].

The design of positively charged metal-inorganic frameworks (MIFs) based on silver cations with polyphosphide linkers demonstrates that targeted host–guest engineering enables the isolation of anionic defects within nanochannels through the Coulombic and geometric complementarity of the subsystems. Positively charged (cationic) framework cross-linked by polyphosphide linkers offer a viable platform for systems with a nuclear-free electron gas. Unlike conventional anionic frameworks, the cationic skeleton exhibits inherent electrostatic affinity for injected electrons acting as anions, while the high conformational flexibility of the polyphosphide chains ensures efficient Coulombic relaxation of nanoscale voids [56].

The synthesis of a canonical oxide-salt superionic conductor with the general form Ag_6_I_4_WO_4_ demonstrates diverse pathways for engineering matrices with high room-temperature ionic conductivity within a partially amorphous inorganic host. The established thermodynamic and chemical stability of this tungstate compound in aggressive oxidizing halogen environment highlights its potential as an ultra-stable matrix for the thresholdless injection and long-term confinement of a high-density electride Fermi gas [57].

The foundational physicochemical principles of carrier transport in solid electrolytes are comprehensively summarized in monographs on solid-state ionics [58,59]. It is important to note that this study addresses only a small subset of known solid-state ionic conductors. A vast unexplored chemical space of multicomponent latent matrices remains beyond the scope of the present work. The primary quantum-chemical criterion—the ionization potential difference (Δ*φ*_HG_ > 0)—defines the fundamental thermodynamic boundaries of this domain. Meanwhile, generalized lattice compliance descriptors (including Cauchy pressure tensor analogs for low-symmetry systems, and the elasto-dielectric invariants of hybrid media) serve as key prescreening markers for achieving record-high concentrations of denucleated electrons.

This study overcomes the inherent limitations of static AI-driven screening by shifting the search for ambient-pressure electrides toward dynamic heterointerface engineering. Consequently, the primary scientific objective becomes the execution of direct in situ experiments coupled with a comprehensive investigation of the transport properties and operational regimes of dynamically forming non-stoichiometric electrides. Their unique properties enable applications in electron emission, heterogeneous catalysis, and various quantum-electronic effects, emerging upon reaching Fermi gas densities of 10^21^–10^22^ cm^−3^.

## 4. Conclusions

In this work, we present a comprehensive description of an alternative pathway for screening dynamically forming electrides from an original perspective. We introduce novel screening descriptors and demonstrate the universal possibilities of electride formation within inorganic and organic solid electrolyte matrices under ambient conditions of 1 atm and 300 K.

For the first time, we have generalized and formulated the principles for the targeted screening, design, and controlled synthesis of electrides stable at room temperature and atmospheric pressure. These findings, initiated by the development of nanoionics, contribute to this field—the science of fast ion transport and reactivity at the nanoscale.

Experimental data show that the interstitial electron density in non-stoichiometric silver and copper (Ag/Cu)-based halides reaches values exceeding 3 × 10^21^ cm^−3^—well above the Mott critical density *n_c_* = (0.25/*a_B_*^∗^)^3^, which for moderate effective masses (m^∗^ ∼ m_e_) and dielectric constants typical of the host matrices (*ε* ∼ 5–10) falls in the range of 10^20^–10^21^ cm^−3^. At such densities, electron wavefunction overlap is no longer perturbative, placing the system in the degenerate regime, consistent with degenerate Fermi liquid behavior, pending direct transport confirmation. In the heavy-fermion regime (*m*^∗^ ≫ *m_e_*), the Mott threshold shifts to higher densities—but a large effective mass itself arises from hybridization and delocalization of interstitial electrons, presupposing that the system is already on the degenerate side of the transition. This interpretation is supported by three convergent lines of indirect evidence. First, the modified Smakula analysis (Equations (4) and (5)) yields interstitial electron densities far exceeding the Mott threshold; moreover, Smakula’s formula is expected to underestimate the true density at such high concentrations, as it assumes non-interacting centers and does not account for wave function overlap near the Mott regime.

Second, in these systems, the increase in interstitial electron density is accompanied by a simultaneous increase in guest ion concentration—a coupled electron–ion degree of freedom not addressed in existing degenerate electron gas theory; strong electron–ion scattering in this regime may lead to large effective masses (heavy-fermion-like behavior) so that even future transport measurements showing low mobility would not contradict the degenerate interpretation. Third, theoretical studies predict characteristic electronic phase transitions when interstitial electron density exceeds the Mott threshold; the phase transitions observed in this work are qualitatively consistent with these predictions. The concentration estimate relies on Smakula’s formula for *F*-centers, which yields underestimated values at such high concentrations. Furthermore, the data were obtained based on calculations of atomic ratios, taking into account the thicknesses of the host matrix and the films deposited onto it. Direct evidence of the elevated electron density is provided by a fundamental transformation of the optical absorption spectra associated with the synthesis of new phases and an increase in the lattice parameter (the appearance of reflections corresponding to larger interplanar spacings).

Fundamental screening descriptors for dynamically forming electrides have been identified: the quantum–chemical inequality Δ*φ*_HG_ > 0, ensuring barrierless penetration of guest species into the *H*-matrix, and the elastic lability descriptor of the *H*-matrix, *П*_Lab_ = (*C*_12_ − *C*_44_)/*B* > 0.35, which ensures facile incorporation of interstitial electrons and guest ions followed by lattice relaxation.

The use of general descriptors and tensor analogs of *П*_Lab_ shifts materials science screening from static brute force searches of massive compound libraries to multidimensional chemical–technological forecasting of stable electride phases dynamically forming in solid-state ionic conductors under non-equilibrium conditions.

The prospects for screening new matrix classes for dynamically forming electrides have been analyzed, confirming the feasibility of extending these results into broader series of latent hosts. Promising candidates for void engineering and interstitial electron localization include porous metal–organic frameworks (MOFs), metal–inorganic frameworks (MIFs), organic ionic plastic crystals (OIPCs), polymer–pyridinium chains (PVP), and oxygen-free superionic glasses.

The concept of inorganic electrides has evolved over nearly four decades. Notably, the first experimental observation of a phenomenon now understood as residing at the threshold of the electride regime at a solid-electrolyte/metal nanoheterointerface was reported by Despotuli in 1997 [19] within the framework of nanoionics—a field whose term and conception had been introduced by Despotuli and Nikolaichik several years earlier [38]. In that study, vacuum deposition of samarium onto RbAg_4_I_5_ films at 293 K produced a nonstoichiometric phase with a record-high concentration (~3 × 10^20^ cm^−3^) of electron centers in anion vacancies, described at the time as *F*-centers. The phase persisted for several days in air before gradually bleaching as the guest metal oxidized—a phenomenology identical to that reported in the present work. However, these results were not interpreted within the electride framework, which had not yet been established for inorganic systems. Attempts to reproduce the effect with aluminum in the same study failed—a result now attributable to the insufficient vacuum (~10^−6^ Torr) used at the time, under which aluminum oxidizes in the deposition chamber before reaching the solid-electrolyte surface. The high-vacuum conditions employed in the present work resolve this artifact and demonstrate electride formation with aluminum and other guest metals.

The electride concept was formalized with the discovery of C12A7:e^−^ by Hosono [7] the first room-temperature-stable inorganic electride in which anionic electrons occupy crystallographic cages in a mayenite framework. Subsequent work extended the electride concept to two-dimensional layered systems (Ca_2_N, 2013; Y_2_C, 2015–2016), intermetallic compounds and interstitial subcarbides exhibiting topological and magnetic properties (Gd_2_C, 2011–2020; LaScSi, 2017) [60,61,62]. By 2021, a comprehensive review by Hosono and Kitano [63] catalogued electrides spanning 0D to 3D, crystalline to amorphous, and bulk to surface forms—yet all known electrides were bulk phases synthesized through high-temperature reduction, metal melting, or high-pressure methods. The subsequent five years witnessed rapid expansion: high-throughput computational screening and machine learning frameworks identified hundreds of new electride candidates, while experimental studies demonstrated magnetic ordering, topological Fermi arcs, superconductivity, and water-stable electride phases.

A 2026 review by Wang et al. provides an up-to-date synthesis of this progress [1]. Notably, however, even this most recent systematic account describes the domain without dynamically forming electrides.

This work builds on the earlier nanoionics observations of Despotuli [19], validated across multiple solid-electrolyte matrices as detailed above. Through the systematic reinterpretation of this 1997–2003 dataset, we advance a new nanoionics–electride paradigm, framing it as a primary contribution that bridges fast ion kinetics and interstitial electron localization. Within this framework, it is demonstrated how the magnitude of the fundamental control parameter—the first ionization potential difference—actively dictates the thermodynamic evolution pathway and phase-space attractors of dynamically forming electrides, expanding the concept beyond the bulk and thin-film paradigms into the regime of nanoscale interfacial self-organization—a regime originally opened up by the nanoionics approach [38].

By establishing a generalizable framework for expanding the chemical space of electrides beyond traditional bulk synthesis, this work bridges electride chemistry and solid-state ionics. The demonstration that magnetic guest ions (e.g., Mn^2+^) can be incorporated alongside non-magnetic electride-forming guests opens a further pathway: interfacial electride-based spintronics, where quasilocalized electrons in ionic nanocavities may mediate magnetic exchange—a direction inaccessible to conventional bulk electride synthesis. The main distinctions between the historical experimental baseline [19,20,21,22,23,24] and new theoretical discoveries and contributions are summarized in Table 1.

## Figures and Tables

**Table 1:** **Hierarchical breakdown of historical experimental baseline vs. new theoretical discoveries and contributions**.

Conceptual Level and Descriptor	Refs. [19,20,21,22,23,24] Baseline	New Theoretical Contribution Here
**I. Core Discovery** (Physical Qualification of Systems)	Phenomenological observation of *F*-centers with high concentrations (>3 × 10^20^ cm^−3^) in Ag/Cu halide nanosystems with >10 G-metals, interpreted as isolated point defects without a unified physical framework.	Theoretical discovery: the identification and physical qualification of a new class of dynamically forming electrides based on matrices of solid-state ionic conductors under ambient conditions (300 K, 1 atm), where the interface spontaneity driving force corresponds to a qualitative drop in Gibbs free energy (Δ*G* < 0). The process is placed within the electride classification of [1] as a third synthesis route—a fundamental alternative to high-pressure and static-screening approaches. This work establishes a generalizable framework for expanding the chemical space of electrides beyond traditional fields and creates synergy between the domains of electrides–berthollides, solid-state ionics/nanoionics, and spintronics.
**II.Predictive Screening** (Materials Selector)	Empirical descriptor formulated as specific pairwise inequalities (e.g., φ_Mn_ < φ_Ag_, φ_Fe_ > φ_Ag_) establishing necessary conditions for cation exchange in Ag/Cu halide systems [21]; not generalized to a universal form or validated as an operational selector across the full *G*-metal space.	Elevation of the pairwise ionization potential inequalities to a formal universal descriptor Δφ_HG_ > 0 (Equation (1)), validated across ~100 independent experiments with >10 *G*-metals, including negative controls (Fe, Ni, Au: Δ*φ*_HG_ < 0 → no reaction). The magnitude of this control parameter actively dictates the non-equilibrium evolution pathway and multi-stable trajectories of dynamically forming electrides at the heterointerface, operating via interface ion-exchange dynamics in structurally disordered conductors rather than through static lattice geometry.
**III. Phase Discrimination** (Physical Status Criteria)	Phenomenological assignment to isolated point defects (*F*-centers); no electride framework or interstitial electron paradigm considered.	(1) Physical explanation of the barrierless interfacial mechanism: in structurally disordered superionic matrices, the boundary layer is already disrupted by space-charge effects (K.Lehovec, a basic fact of nanoionics) prior to *G*-metal deposition; static Madelung invariants are destroyed from the outset, so the gas-phase descriptor Δ*φ*_HG_ > 0 functions as an operationally sufficient energy selector without a static lattice barrier. (2) Introduction of normalized Cauchy pressure *Π*_Lab_ as a quantitative orientation guide for future automated screening—not a universal law—grounded in the contrast between rigid alkali–halide matrices (R.Pohl school, *Π*_Lab_ ≈ 0, *F*-centers limited to ~10^17^ cm^−3^) and superionic conductors (RbAg_4_I_5_: 0.379, AgI: 0.614), where excess cationic positions sustain ionic motion and enable electride formation.
**IV. Metrological Analysis** (Quantification of Densities)	Concentration estimates based on the classical Smakula equation for isolated, non-interacting point defects (Smakula, 1930 [25]).	Two-stage methodology: (1) the linear Smakula equation is shown to remain valid up to ~10^20^ cm^−3^, serving as a high-precision metrological probe for tracking oscillator strength degradation and early deviations from the Mott criterion; (2) for the extreme macro-doping regime (>10^21^ cm^−3^), a modified Smakula relation with density-dependent correction *Γ*(*N*) (Equations (4) and (5)) is introduced as a prospective theoretical framework to account mathematically for wavefunction overlap at high anion–electron densities. Cross-validation between the two stages provides order-of-magnitude estimates > 10^21^ cm^−3^.
**V. Materials Synthesis** (Thermodynamic Pathways)	No alternative synthesis route proposed; experiments conducted within conventional preparation frameworks for Ag/Cu halide systems.	A third synthesis route exploiting compliant halide superionic conductors as dynamically reconfigurable matrices at 293 K through interfacial ion exchange dynamics (Section 3.2).
**VI. Magneto-Optics** (Spin–Orbit Tuning Fields)	No magneto-optical characterization performed in Refs. [19,20,21,22,23,24]; all optical measurements restricted to zero magnetic field.	First report of giant, strictly reversible Zeeman splitting of the E_1_ interface exciton in Ag_2_MnI_4_ (*g* ≈ 890, *B* = 0.85 T, 293 K; archival data obtained in 2003, previously unpublished). Two-attractor model for the AgI/Mn system: berthollide trajectory ↔ ordered Ag_2_MnI_4_ magnetic sublattice. Opens up the chemical space to room temperature electride spintronics, including multi-component and high-entropy electride–berthollide frameworks beyond the demonstrated Ag-Mn system.

## Data Availability

The original contributions presented in this study are included in the article. Further inquiries can be directed to the corresponding author.
